# Automated AI-Driven CT Quantification of Lung Disease Predicts Adverse Outcomes in Patients Hospitalized for COVID-19 Pneumonia

**DOI:** 10.3390/diagnostics11050878

**Published:** 2021-05-14

**Authors:** Marie Laure Chabi, Ophélie Dana, Titouan Kennel, Alexia Gence-Breney, Hélène Salvator, Marie Christine Ballester, Marc Vasse, Anne Laure Brun, François Mellot, Philippe A. Grenier

**Affiliations:** 1 Department of Medical Imaging, Foch Hospital, 92150 Suresnes, France; ml.chabi-charvillat@hopital-foch.com (M.L.C.); opheliedana75@gmail.com (O.D.); gence-breney.alexia@hotmail.fr (A.G.-B.); al.brun@hopital-foch.com (A.L.B.); f.mellot@hopital-foch.com (F.M.); 2 Department of Clinical Research and Innovation, Foch Hospital, 92150 Suresnes, France; t.kennel@hopital-foch.com; 3 Department of Pneumology, Foch Hospital, UFR Santé Simone Veil UVSQ Paris-Saclay University, 92150 Suresnes, France; h.salvator@hopital-foch.com; 4 Department of Emergency Medicine, Foch Hospital, 92150 Suresnes, France; mc.ballester@hopital-foch.com; 5 Department of Clinical Biology, Foch Hospital, 92150 Suresnes, France; m.vasse@hopital-foch.com; 6 INSERM, UMRS 1176, Paris-Saclay University, 94270 Le Kremlin-Bicêtre, France

**Keywords:** COVID-19, pneumonia, quantitative CT, artificial intelligence, outcome prediction, multivariate analysis

## Abstract

The purpose of our work was to assess the independent and incremental value of AI-derived quantitative determination of lung lesions extent on initial CT scan for the prediction of clinical deterioration or death in patients hospitalized with COVID-19 pneumonia. 323 consecutive patients (mean age 65 ± 15 years, 192 men), with laboratory-confirmed COVID-19 and an abnormal chest CT scan, were admitted to the hospital between March and December 2020. The extent of consolidation and all lung opacities were quantified on an initial CT scan using a 3D automatic AI-based software. The outcome was known for all these patients. 85 (26.3%) patients died or experienced clinical deterioration, defined as intensive care unit admission. In multivariate regression based on clinical, biological and CT parameters, the extent of all opacities, and extent of consolidation were independent predictors of adverse outcomes, as were diabetes, heart disease, C-reactive protein, and neutrophils/lymphocytes ratio. The association of CT-derived measures with clinical and biological parameters significantly improved the risk prediction (*p* = 0.049). Automated quantification of lung disease at CT in COVID-19 pneumonia is useful to predict clinical deterioration or in-hospital death. Its combination with clinical and biological data improves risk prediction.

## 1. Introduction

The SARS-COV-2 is currently responsible for a worldwide pandemic that may lead to severe viral pneumonia with critical complications, including acute respiratory failure requiring admission in an intensive care unit (ICU) for mechanical ventilation or high flow oxygenation. Among patients hospitalized for COVID-19, 14–30% will require admission to an ICU, and 20–33% of them will die [1,2,3,4]. Identification of patients at risk for severe COVID-19 is critical in order to deliver proper care and to optimize requirements for limited ICU resources [5]. Even if the staging of patients upon their hospital admission is mainly based on risk scores that combine several clinical and biological biomarkers [6,7,8,9,10], the role of imaging may be focused on an estimation of the lung disease extent from CT scans. CT findings can indicate disease stage [11,12,13] and predict adverse outcomes [14,15,16]. Patients with severe COVID-19 pneumonia tend to have more opacities, and the increase in lung consolidation that occurs most often later in the disease tends to be associated with more critical illness [17,18]. Despite two studies showing that lung disease extent in COVID-19 pneumonia assessed by visual scoring correlates with clinical disease severity [19,20], visual estimation of disease extent even done by experimented radiologists may be a source of variability. Artificial Intelligence (AI) software developed to help radiologists in the quantification of lung involvement in COVID-19 may overcome this limitation [21]. Some investigators developed their own AI system for accurate quantitative measurements and prognosis of COVID-19 pneumonia using CT [22,23,24]. However, their systems were trained and validated on data from a single-center or few centers located in the same geographical area. This may question the reproducibility of AI system performances when applied in other regions or countries.

We hypothesized that an AI-based software trained, validated, and tested on an international basis, providing a 3D quantification of lung disease on initial CT scan performed in patients hospitalized for COVID-19 pneumonia could predict clinical deterioration requiring transfer into an intensive care unit or death, and in addition, could provide an incremental prognostic value when the quantitative CT-measures are associated with clinical and biological parameters.

## 2. Materials and Methods

### 2.1. Study Design

This retrospective study performed in a tertiary Parisian center was approved by the Ethics Committee of Foch Hospital, affiliated to the Versailles Saint-Quentin University, and the requirement for informed consent was waived for fully anonymized data analysis.

Consecutive patients hospitalized for COVID-19 pneumonia (from 15 March to 15 December 2020), and having an abnormal chest CT scan performed at admission or within the 48 h after admission in Foch Hospital, Suresnes, France, were included. Patient charts were reviewed in terms of age, sex, and comorbidities (diabetes, systemic hypertension, COPD, heart disease, chronic renal disease, and immunodeficiency). We recorded the delay between the beginning of symptoms and the first CT scan performed at admission or within the 48 h after admission and collected all biological data, including inflammation indicators that have been previously reported to be associated with the prognosis of COVID-19 [10]. Medical records were reviewed for outcome assessment within one month following the CT examination; the severe outcome was defined by transfer to an intensive care unit (ICU) to receive an oxygen flow rate of 15 L/min or higher, the need for mechanical ventilation, or patient death.

### 2.2. CT Scans

Images acquisitions were performed on a Siemens CT scanner (Edge 1; Siemens Healthineers, Forchheim, Germany). All patients were scanned in a supine position during an inspiratory breath-hold. Contiguous 0.6 mm thick axial images were reconstructed with high resolution and soft tissue kernels using an iterative reconstruction algorithm. Contrast material injection was used in patients because of suspicion of pulmonary embolism associated with COVID-19 infection. Parameters used for scans without intravenous contrast included a peak x-ray tube voltage of 120 or 140 kVp, automatic tube current modulation (DLP: 100–140 mGy.cm), and a slice thickness of 0.6 mm. The protocol for contrast-enhanced scans included a peak x-ray voltage of 120 kVp, automated tube current modulation (DLP: 25O–350 mGy.cm), and a slice thickness of 0.6 mm. A total of 60 mL iodinated contrast material was injected intravenously at a rate of 4ml/sec, followed by 30 mL of saline chaser at a flow rate of 4 mL/s.

### 2.3. Automatic Quantification of Lung Disease Extent

CT scans of patients hospitalized were analyzed using software providing automatic segmentation and quantification of lungs, lobes, and affected areas in the lung parenchyma. This AI solution (AI-Rad Companion Research CT Pneumonia Analysis) was developed by Siemens Healthineers to assess quantitatively the extent and severity of lung disease in patients with COVID-19 pneumonia [25]. The software was trained and validated with CT scans of COVID-19 pneumonia from two European countries, Canada, and the USA. It outputs regions of abnormality demarcated in a three-dimensional CT scan and two combined measures of lung disease extent. The first measures indicate the overall disease spread relative to the volume of the lungs, while the second measures are lobe-wise according to automatic segmentation of peripheral, mediastinal, and fissural limits of the lobes. The global extent of lung disease is measured as the percentage of lung volume occupied by all opacities including ground glass, crazy paving, and consolidation, followed by the percentage of lung volume just occupied by high dense opacities corresponding to consolidation and determined by using an empirical density threshold (−200 HU). The results are provided in 1 min and 40 s.

### 2.4. Statistical Analysis

Receiver operating characteristics (ROC) was plotted, and the area under the curve (AUC) was calculated with 95% confidence intervals for the prediction of severe outcome by the percentages of lung volume occupied by all opacities and by high-density opacities only. Discriminant thresholds were obtained using the Youden index (Sensitivity + Specificity − 1). Univariate logistic regression models were performed, followed by multivariate models using the variables associated with the severe outcomes (*p* value < 0.2).

The final model was obtained using the stepwise descending procedure. All tests were bilateral with a 5% degree of significance. Statistical analyses were performed with the SAS 9.4 software.

## 3. Results

### 3.1. Patients Characteristics

The final study population included 323 patients (mean age 65 yrs (±15), 192 men) with confirmed COVID-19 infection by a positive RT-PCR (real-time fluorescence polymerase chain reaction) on nasopharyngeal tract specimens. The mean time from self-reported onset of symptoms to hospital admission was 8.33 (±5.17) days.

The severe outcome (transfer in ICU or death) occurred in 85 (26.3%) patients: 54 (16.7%) were admitted to an intensive care unit; 26 (8%) received high flow oxygen only, and 28 (8.6%) required mechanical ventilation. Finally, 38 (11.8%) patients died, among which seven died during the first four days of hospitalization. The remaining patients (*n* = 238; 73.7%) did not require critical care and had been discharged alive one month after admission. Patients who experienced deterioration or death were not older (66.48 ± 14.45) than those who did not (63.77 ± 15.21) (*p* = 0.15). The male/female ratio was significantly higher in patients who presented severe outcomes than those who did not (*p* = 0.02). The mean time from self-reported onset of symptoms to CT scan was not statistically significant between patients who were transferred in ICU or died (7.73 ± 5.21 days) and those who did not (8.54 ± 5.15 days) (*p* = 0.24). Among comorbidities, the only ones significantly associated with severe outcomes were diabetes and heart disease (Table 1). Among laboratory results, only C-reactive protein, procalcitonin, lactate dehydrogenase, neutrophils count, and neutrophils/lymphocytes ratio were significantly associated with severe outcomes (Table 1).

### 3.2. Chest CT Findings

A total of 184 (57%) patients underwent non-contrast chest CT, while the remaining 139 (43%) underwent CT angiography for exclusion of pulmonary embolism. The presence of clots in segmental or subsegmental pulmonary arteries was noted in 13 (9.3%) patients, among which 11 did not present clinical deterioration or die (*p* = 0.33).

**Figure 1:**
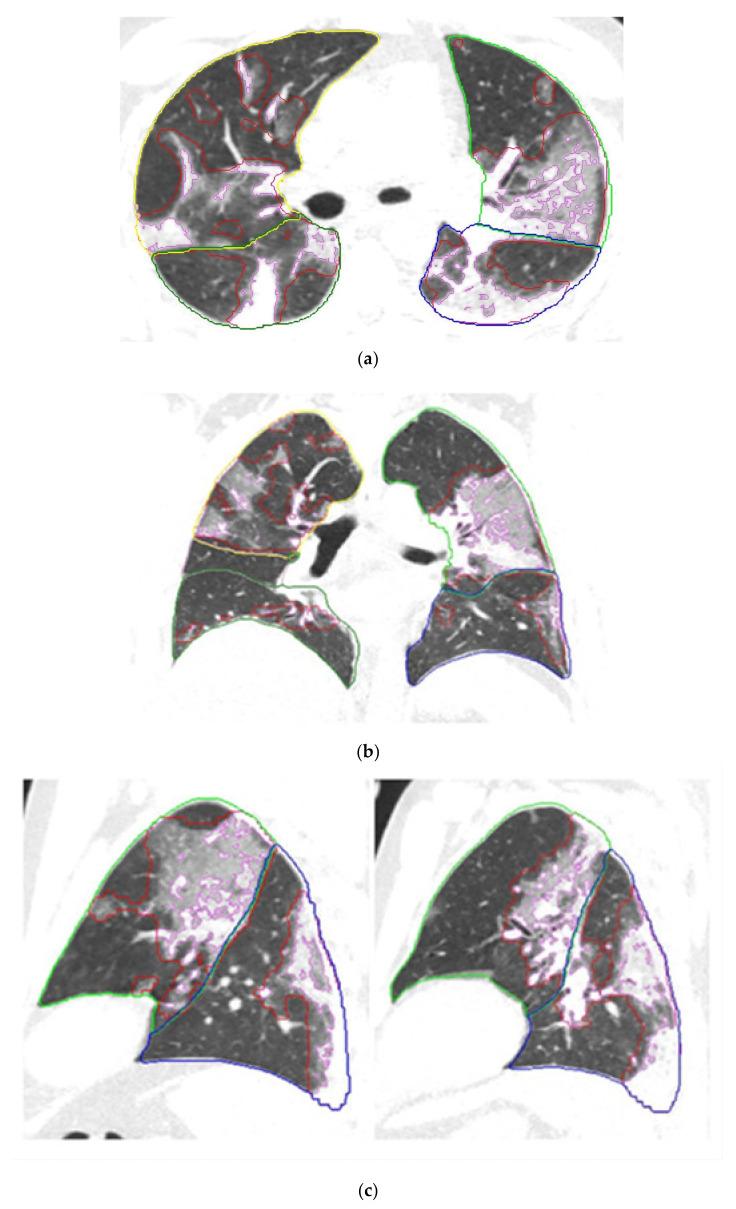
Chest CT scan performed the day of hospital admission in a 46 yo male COVID-19 patient. Axial (**a**), Coronal (**b**), and Sagittal (**c**) reformatted CT images after application of the AI-driven software. The interfaces between the pleura and lung parenchyma were automatically segmented by the software. The limits of the lobes are marked with different colors: yellow for the right upper lobe, green for the left upper lobe, dark green for the right lower lobe, and blue for the left lower lobe. The red marks represent the contours of lung opacities segmented by the software, and the purple marks limit the consolidation areas. Automatic measures of the extent of consolidation and all lung opacities were 13% and 39% of lung volume respectively. This patient had diabetes and presented increased C-reactive protein level and neutrophils/lymphocytes ratio. After 3 days of hospitalization, because of clinical deterioration, the patient was transferred to the intensive care unit for high-flow oxygen therapy.

Quantitative lung features on CT are summarized in Table 1. Patients with deterioration or death had a higher extent of both all opacities (37.48 ± 21.47% of lung volume) and higher extent of consolidation (8.87 ± 8.22% of lung volume) on CT scan performed at admission or within 48 h following admission, compared to patients who did not require critical care or were discharged alive (22.63 ± 17.01% and 5.00 ± 5.64%, respectively) (*p* < 0.001). Figure 1 shows a representative case of CT findings automatically segmented and quantified by the AI-based software.

### 3.3. Predictors of Clinical Deterioration or Death

The area under the curve (AUC) to predict deterioration or death, when using the volume percentage of all opacities, was 0.70, with 81% sensitivity and 54% specificity obtained at the optimal threshold (Youden index = 33). When using high densities only expressing consolidation, the AUC was 0.68, with 46% sensitivity and 87% specificity at the optimal threshold (Youden index = 2). In other words, 33% or more of lung volume occupied by lung opacities provided an 81% sensitivity, and 2% or less of lung volume occupied by consolidation provided an 87% specificity to predict clinical deterioration or death.

In a multivariate analysis including clinical, biological, and quantitative CT parameters (Table 2), both volume percentages of all opacities (OR, 2.70; 95% CI: 1.49–4.88, *p* < 0.001) and consolidation (OR, 4.08; 95% CI: 1.90–8.78, *p* < 0.001) were independent predictors of deterioration or death, as were diabetes, history of cardiac disease, serum C-reactive protein, and neutrophils-lymphocytes ratio.

The performance of quantitative CT measures in risk prediction was not significantly different from those of clinical (AUC: 0.62) and biological (AUC: 0.67) parameters isolated but the association of all components increased significantly the risk prediction (AUC: 0.79), (*p* = 0.049).

## 4. Discussion

In this monocentric study of patients hospitalized for COVID-19 pneumonia in a Parisian center, we found that extent of lung infiltration assessed on CT scan performed at admission or within the first 48 h after admission independently predicts the risk of clinical deterioration or in-hospital death. Ground glass opacities are considered to represent the early exudative phase of pneumonia, secondarily progressing to consolidation with intra-alveolar organization, fibrotic proliferation, and alveolar collapse [26]. Chest CT performed within the first five days of symptom onset typically demonstrates a ground-glass predominant pattern, followed by increasing consolidative changes for up to 14 days [11,12,27]. Hence, consolidation represents the peak stage of COVID-19 pneumonia with intense inflammatory changes associating organization pneumonia and alveolar damage, corresponding to the phase of infection during which the patients are the most critically ill [10].

As prior investigators did [28], we showed that the extent of consolidation independently predicts adverse outcomes (ICU admission or in-hospital death) with a good specificity despite a modest sensitivity. Low sensitivity of consolidation is easily explained as CT assessment was performed at the beginning of hospitalization, at an early stage of the disease characterized by a CT pattern with predominant ground-glass opacities. Our reported prognostic values for CT scan-based models (0.68–0.70 AUC) are lower than some reported in previously published studies (0.75–0.85 AUC) using AI-based quantitative analysis of CT scans images for prognosis [22,23,24,28]. We hypothesize this could be due to the use of differences in outcome definitions and patients’ clinical characteristics (age, severity at admission, etc.).

Compared to a radiologist’s reporting with visual quantitative estimation of lesions, there are several advantages to capturing CT scan information through 3D AI-based software. Good reproducibility is a key element for imaging biomarkers, and visual inspection of images introduces variability that can hinder its clinical application [23]. In addition, AI analysis of radiologic images reduces the radiologist reading time. Furthermore, as demonstrated by Lassau et al., prognostic scores obtained with AI analysis are more predictive of severity than a quantitative visual scoring of disease extent performed by radiologists [22].

In our study, we also showed that CT-derived quantitative lung measures have incremental prognostic value when used in combination with clinical and biological parameters. It is established that the presence and number of comorbidities predict clinical outcomes in patients with COVID-19 [1]. Specifically, older age, chronic cardiac or pulmonary disease, hypertension, diabetes, and chronic kidney disease all confer an increased risk of in-hospital mortality [9,10]. Surprisingly, in our cohort, the patient age was not predictive of clinical deterioration or death. Among comorbidities observed in our cohort, only heart disease and diabetes were associated with a poorer outcome. Hypertension was frequently present in our hospitalized COVID-19 patients; however, the percentage of patients suffering from hypertension was not different between those who presented clinical deterioration or died and those who did not.

Among biological variables, inflammatory biomarkers, C-reactive protein, lactate dehydrogenase (LDH), procalcitonin levels at admission were shown to associate with a greater risk of worse clinical course [10,29,30,31,32,33]. In accordance with Lassau et al. [22], we found an association of clinical severity with increased neutrophils count and neutrophils-lymphocytes ratio. Few studies have combined clinical and biological data with chest CT for prognostication in COVID-19 pneumonia [14,21,22,23]. Like previous investigators, we demonstrate here that a model integrating AI-based CT scan information and clinical and biological parameters improves the prognosis performance of the score.

The main limitation of our study is the retrospective design. As a result, some clinical and biological data were missing. Particularly lactate dehydrogenase and procalcitonin were measured only in 60% and 47% of our patients, respectively. This explains why we did not include these biomarkers in our prediction models despite both were significantly different between patients who experienced deterioration or death compared to those who did not. Troponin, creatinine kinase, interleukine-6, platelet count, total and conjugated bilirubin were not uniformly available and thus not included in our risk prediction models. Furthermore, the repetition of biological parameters during the first hours during the stay in the emergency department before admission were not performed or considered, but we know from a study by Solimando et al. short-term increases in the neutrophil-to-lymphocyte ratio and urea-to-creatinine ratio were independent predictors of the intensive care unit [34].

Iodinated contrast injection performed in 43% of our patients was also a potential limitation in our study. Despite there was no significant difference in the percentage of patients receiving contrast material on CT scan between patients who experienced deterioration or death and those who did not, this may have induced a change in lung density measurement within areas of lung infiltration, leading to a change in selecting areas of high densities treated as consolidation. Finally, the relatively small number of patients included in the study is also a limitation, but it reflects a monocentric experience of the COVID-19 epidemic.

## 5. Conclusions

Quantitative assessment of radiological extent and severity of lung disease using an automatic 3D AI-based software independently can predict clinical deterioration or death in COVID-19 pneumonia. Thus, CT-derived quantitative measures could have prognostic value that may be used in association with clinical and biological independent predictors of clinical deterioration or death and could be useful for clinical risk stratification in patients with COVID-19. However, the present study being retrospective, should be completed by further prospective investigations including a larger population with an exhaustive research of comorbidities and completion of all repeated biological examinations.

## Figures and Tables

**Table 1:** Clinical and Laboratory Characteristics on admission and quantitative CT parameters in univariate logistic regression analysis.

		Clinical Deterioration or Death		
	N	Yes (*n* = 85)	No (*n* = 238)	*p* Value
**Clinical characteristics**				
Age, years	323	66.48 (±14.45)	64.77 (±15.21)	0.15
Male sex: N (%)	323	56 (65.88)	138 (57.98)	0.02
Onset of symptoms	300	7.73 (±5.21)	8.54 (±5.15)	0.24
Hypertension: N (%)	323	39 (45.88)	110 (46.22)	0.96
Diabetes: N (%)	323	35 (41.18)	55 (23.11)	**0.002**
Heart disease: N (%)	323	20 (23.53)	26 (10.92)	**0.005**
COPD: N (%)	323	6 (7.06)	8 (3.36)	0.16
Chronic renal disease: N (%)	323	15 (17.65)	34 (14.21)	0.46
Immunodeficiency: N (%)	323	24 (26.63)	53 (22.75)	0.22
**Laboratory results** (mean ±SD)				
C-reactive protein, mg/L	310	152.10 (±107.67)	94.70 (±73.44)	**<0.001**
Ferritin, ng/mL	233	3185.90 (±11037)	1366.76 (±2856.62)	0.21
D-Dimers, µg/mL	257	3.59 (13.20)	1.75 (±3.87)	0.16
Procalcitonin, ng/mL	156	2.42 (±7.27)	0.44 (±1.12)	**0.006**
LDH, IU/L	194	480.22 (±177.88)	398.89 (±149.63)	**0.004**
Lymphocytes, G/L	317	0.9 (±0.65)	1.00 (±1.08)	0.44
Neutrophils G/L	317	6.6 (±4.25)	5.13 (±2.7)	**0.001**
Neutrophils/Lymphocytes	317	14.38 (±35.38)	7.5 (±7.67)	**0.008**
**CT characteristics**				
IV Contrast material: N (%)	323	36 (42.35)	101 (42.44)	0.99
Volume % of all opacities	323	37.48 (±21.47)	22.63 (±17.01)	**<0.001**
Volume % consolidation	323	8.87 (±8.22)	5.00 (±5.64)	**<0.001**

**Table 2:** Association of clinical and CT parameters with risk of clinical deterioration or death in a multivariate logistic regression analysis.

	Model 1 (% All Opacities)		Model 2 (% Consolidation)	
	OR [IC95%]	*p*-Value	OR [IC95%]	*p*-Value
Diabetes	2.30 [1.26–4.22]	0.007	2.31 [1.25–4.25]	0.007
Heart disease	2.79 [1.34–5.81]	0.006	2.78 [1.33–5.80]	0.006
C-reactive protein	1.32 [1.12–1.56]	0.001	1.28 [1.08–1.51]	0.004
Neutrophils/Lymphocytes	1.03 [1.00–1.06]	0.047	1.03 [1.00–1.07]	0.030
% all opacities at CT	2.70 [1.49–4.88]	0.001		
% consolidation at CT			4.08 [1.90–8.78]	<0.001

## Data Availability

No new data were created or analyzed in this study. Data sharing is not applicable to this article.

## References

[1] Richardson S., Hirsch J.S., Narasimhan M., Crawford J.M., McGinn T., Davidson K.W., Barnaby D.P., Becker L.B., Chelico J.D., the Northwell COVID-19 Research Consortium (2020). Presenting Characteristics, Comorbidities, and Outcomes Among 5700 Patients Hospitalized with COVID-19 in the New York City Area. JAMA.

[2] Docherty A.B., Harrison E.M., Green C.A., Hardwick H.E., Pius R., Norman L., Holden K.A., Read J.M., Dondelinger F., Carson G. (2020). Features of 20 133 UK patients in hospital with covid-19 using the ISARIC WHO Clinical Characterisation Protocol: Prospective observational cohort study. BMJ.

[3] Myers L.C., Parodi S.M., Escobar G.J., Liu V.X. (2020). Characteristics of Hospitalized Adults With COVID-19 in an Integrated Health Care System in California. JAMA.

[4] Wu C., Chen X., Cai Y., Xia J., Zhou X., Xu S., Huang H., Zhang L., Zhou X., Du C. (2020). Risk Factors Associated With Acute Respiratory Distress Syndrome and Death in Patients With Coronavirus Disease 2019 Pneumonia in Wuhan, China. JAMA Intern. Med..

[5] Phua J., Weng L., Ling L., Egi M., Lim C.M., Divatia J.V., Shrestha B.R., Arabi Y.M., Ng J., Gomersall C.D. (2020). Intensive care management of coronavirus disease 2019 (COVID-19): Challenges and recommendations. Lancet Respir Med..

[6] Liang W., Liang H., Ou L., Chen B., Chen A., Li C., Li Y., Guan W., Sang L., Lu J. (2020). Development and Validation of a Clinical Risk Score to Predict the Occurrence of Critical Illness in Hospitalized Patients With COVID-19. JAMA Intern. Med..

[7] Ji D., Zhang D., Xu J., Chen Z., Yang T., Zhao P., Chen G., Cheng G., Wang Y., Bi J. (2020). Prediction for Progression Risk in Patients With COVID-19 Pneumonia: The CALL Score. Clin. Infect. Dis. Off. Publ. Infect. Dis. Soc. Am..

[8] Knight S.R., Ho A., Pius R., Buchan I., Carson G., Drake T.M., Dunning J., Fairfield C.J., Gamble C., Green C.A. (2020). Risk stratification of patients admitted to hospital with covid-19 using the ISARIC WHO Clinical Characterisation Protocol: Development and validation of the 4C Mortality Score. BMJ.

[9] Wynants L., Van Calster B., Collins G.S., Riley R.D., Heinze G., Schuit E., Bonten M.M.J., Dahly D.L., Damen J.A.A., Debray T.P.A. (2020). Prediction models for diagnosis and prognosis of covid-19 infection: Systematic review and critical appraisal. BMJ.

[10] Zhou F., Yu T., Du R., Fan G., Liu Y., Liu Z., Xiang J., Wang Y., Song B., Gu X. (2020). Clinical course and risk factors for mortality of adult inpatients with COVID-19 in Wuhan, China: A retrospective cohort study. Lancet.

[11] Wang Y., Dong C., Hu Y., Li C., Ren Q., Zhang X., Shi H., Zhou M. (2020). Temporal Changes of CT Findings in 90 Patients with COVID-19 Pneumonia: A Longitudinal Study. Radiology.

[12] Bernheim A., Mei X., Huang M., Yang Y., Fayad Z.A., Zhang N., Diao K., Lin B., Zhu X., Li K. (2020). Chest CT Findings in Coronavirus Disease-19 (COVID-19): Relationship to Duration of Infection. Radiology.

[13] Pan F., Ye T., Sun P., Gui S., Liang B., Li L., Zheng D., Wang J., Hesketh R.L., Yang L. (2020). Time Course of Lung Changes at Chest CT during Recovery from Coronavirus Disease 2019 (COVID-19). Radiology.

[14] Colombi D., Bodini F.C., Petrini M., Maffi G., Morelli N., Milanese G., Silva M., Sverzellati N., Michieletti E. (2020). Well-aerated Lung on Admitting Chest CT to Predict Adverse Outcome in COVID-19 Pneumonia. Radiology.

[15] Yuan M., Yin W., Tao Z., Tan W., Hu Y. (2020). Association of radiologic findings with mortality of patients infected with 2019 novel coronavirus in Wuhan, China. PLoS ONE.

[16] Lyu P., Liu X., Zhang R., Shi L., Gao J. (2020). The Performance of Chest CT in Evaluating the Clinical Severity of COVID-19 Pneumonia: Identifying Critical Cases Based on CT Characteristics. Investig. Radiol..

[17] Tabatabaei S., Talari H., Moghaddas F., Rajebi H. (2020). CT Features and Short-term Prognosis of COVID-19 Pneumonia: A Single-Center Study from Kashan, Iran. Radiol. Cardiothorac. Imaging.

[18] Yang R., Li X., Liu H., Zhen Y., Zhang X., Xiong Q., Luo Y., Gao C., Zeng W. (2020). Chest CT score: An imaging tool for assessing svere COVID-19. Radiol. Cardiothorac. Imaging.

[19] Li K., Fang Y., Li W., Pan C., Qin P., Zhong Y., Liu X., Huang M., Liao Y., Li S. (2020). CT image visual quantitative evaluation and clinical classification of coronavirus disease (COVID-19). Eur. Radiol..

[20] Cheng Z., Qin L., Cao Q., Dai J., Pan A., Yang W., Gao Y., Chen L., Yan F. (2020). Quantitative computed tomography of the coronavirus disease 2019 (COVID-19) pneumonia. Radiol. Infect. Dis..

[21] Gieraerts C., Dangis A., Janssen L., Demeyere A., De Bruecker Y., De Brucker N., van den Bergh A., Lauwerier T., Heremans A., Frans E. (2020). Prognostic Value and Reproducibility of AI-assisted Analysis of Lung Involvement in COVID-19 at Low-Dose Submillisievert Chest CT: Sample Size Implications for Clinical Trials. Radiol. Cardiothorac. Imaging.

[22] Lassau N., Ammari S., Chouzenoux E., Gortais H., Herent P., Devilder M., Soliman S., Meyrignac O., Talabard M.P., Lamarque J.P. (2021). Integrating deep learning CT-scan model, biological and clinical variables to predict severity of COVID-19 patients. Nat. Commun..

[23] Chassagnon G., Vakalopoulou M., Battistella E., Christodoulidis S., Hoang-Thi T.N., Dangeard S., Deutsch E., Andre F., Guillo E., Halm N. (2021). AI-driven quantification, staging and outcome prediction of COVID-19 pneumonia. Med. Image Anal..

[24] Zhang K., Liu X., Shen J., Li Z., Sang Y., Wu X., Zha Y., Liang W., Wang C., Wang K. (2020). Clinically Applicable AI System for Accurate Diagnosis, Quantitative Measurements, and Prognosis of COVID-19 Pneumonia Using Computed Tomography. Cell.

[25] Chaganti S., Grenier P., Balachandran A., Chabin G., Cohen S., Flohr T., Georgescu B., Grbic S., Liu S., Mellot F. (2020). Automated Quantification of CT Patterns Associated with COVID-19 from Chest CT. Radiol. Artif. Intell..

[26] Tian S., Xiong Y., Liu H., Niu L., Guo J., Liao M., Xiao S.Y. (2020). Pathological study of the 2019 novel coronavirus disease (COVID-19) through postmortem core biopsies. Mod. Pathol..

[27] Shi H., Han X., Jiang N., Cao Y., Alwalid O., Gu J., Fan Y., Zheng C. (2020). Radiological findings from 81 patients with COVID-19 pneumonia in Wuhan, China: A descriptive study. Lancet Infect. Dis..

[28] Grodecki K., Lin A., Cadet S., McElhinney P.A., Razipour A., Chan C., Pressman B., Julien P., Maurovich-Horvat P., Gaibazzi N. (2020). Quantitative Burden of COVID-19 Pneumonia at Chest CT Predicts Adverse Outcomes: A Post Hoc Analysis of a Prospective International Registry. Radiol. Cardiothorac. Imaging.

[29] Ruan Q., Yang K., Wang W., Jiang L., Song J. (2020). Clinical predictors of mortality due to COVID-19 based on an analysis of data of 150 patients from Wuhan, China. Intensive Care Med..

[30] Li X., Xu S., Yu M., Wang K., Tao Y., Zhou Y., Shi J., Zhou M., Wu B., Yang Z. (2020). Risk factors for severity and mortality in adult COVID-19 inpatients in Wuhan. J. Allergy Clin. Immunol..

[31] Guo L., Wei D., Zhang X., Wu Y., Li Q., Zhou M., Qu J. (2019). Clinical Features Predicting Mortality Risk in Patients With Viral Pneumonia: The MuLBSTA Score. Front. Microbiol..

[32] Merugu G.P., Nesheiwat Z., Balla M., Patel M., Fatima R., Sheikh T., Kotturi V., Bommana V., Pulagam G., Kaminski B. (2020). Predictors of mortality in 217 COVID-19 patients in Northwest Ohio, United States: A retrospective study. J. Med. Virol..

[33] Ghahramani S., Tabrizi R., Lankarani K.B., Kashani S.M.A., Rezaei S., Zeidi N., Akbari M., Heydari S.T., Akbari H., Nowrouzi-Sohrabi P. (2020). Laboratory features of severe vs. non-severe COVID-19 patients in Asian populations: A systematic review and meta-analysis. Eur. J. Med. Res..

[34] Solimando A.G., Susca N., Borrelli P., Prete M., Lauletta G., Pappagallo F., Buono R., Inglese G., Forina B.M., Bochicchio D. (2020). Short-Term Variations in Neutrophil-to-Lymphocyte and Urea-to-Creatinine Ratios Anticipate Intensive Care Unit Admission of COVID-19 Patients in the Emergency Department. Front. Med..

